# Validated Erythrosin B spectrofluorimetric method for ganciclovir bioanalysis in rabbit plasma following valganciclovir bioconversion and Pharmacokinetic application

**DOI:** 10.1038/s41598-025-32405-z

**Published:** 2025-12-23

**Authors:** Ali Alqahtani, Taha Alqahtani, Adil Alshehri, Ahmed A. Almrasy

**Affiliations:** 1 Department of Pharmacology, College of Pharmacy, King Khalid University, Abha, 62529 Saudi Arabia; 2 Department of Medicine, College of Medicine, King Khalid University, Abha, 62529 Saudi Arabia; 3 Pharmaceutical Analytical Chemistry Department, Faculty of Pharmacy, Al-Azhar University, Cairo, 11751 Egypt

**Keywords:** Ganciclovir, Erythrosin B, Spectrofluorimetry, Pharmacokinetics, Bioanalytical validation, Chemistry, Physics

## Abstract

A novel, environmentally sustainable spectrofluorimetric method was developed and validated for sensitive determination of ganciclovir in rabbit plasma following valganciclovir administration. The method is based on fluorescence quenching of Erythrosin B probe through static complex formation with ganciclovir, as confirmed by comprehensive spectroscopic characterization using UV-visible absorption and fluorescence spectroscopy. Furthermore, temperature-dependent Stern-Volmer analysis revealed static quenching mechanism with Stern-Volmer constants decreasing from 3.77 × 10^5^ to 2.79 × 10^5^ M^− 1^ upon temperature increase from 298 to 313 K. Moreover, thermodynamic studies demonstrated spontaneous, exothermic interactions with negative Gibbs free energy and enthalpy changes, while positive entropy indicated favorable complex formation. Additionally, Job’s method confirmed 1:1 stoichiometry, while semiempirical PM3 quantum mechanical calculations identified halogen bonding and electrostatic interactions as primary binding mechanisms. Experimental conditions were systematically optimized to achieve maximum quenching efficiency at pH 5.0 with 10 µg/mL Erythrosin B concentration and 5-minute reaction time. The method demonstrated excellent analytical performance with good linearity (r^2^ = 0.9991) over 0.05–3.0 µg/mL range and adequate sensitivity for therapeutic monitoring applications. Furthermore, comprehensive validation according to ICH M10 guidelines confirmed method reliability with accuracy ranging from 96.04% to 104.76% and precision below 4.61% RSD across all quality control levels. Subsequently, successful pharmacokinetic application in New Zealand white rabbits provided key parameters including C_max_ of 4.25 µg/mL, half-life of 2.94 h, and AUC_₀₋∞_ of 21.6 µg·h/mL, consistent with published clinical data. Finally, environmental sustainability assessment using four complementary evaluation tools confirmed favorable green analytical chemistry and analytical practicality traits with scores of 0.71, 76%, 70.0, and 65% for AGREE, MoGAPI, BAGI, and CACI, respectively. In conclusion, the developed spectrofluorimetric method offers significant advantages including cost-effectiveness, minimal waste generation, and elimination of derivatization steps, positioning it as an attractive alternative to conventional methods.

**Supplementary Information:**

The online version contains supplementary material available at 10.1038/s41598-025-32405-z.

## Introduction

Cytomegalovirus (CMV) infection represents a significant clinical challenge in immunocompromised patients, particularly affecting solid organ transplant (SOT) and hematopoietic stem cell transplant (HSCT) recipients where it remains one of the most frequent causes of morbidity and mortality^1,2^. Ganciclovir, a synthetic nucleoside analog of guanosine, serves as the first-choice therapeutic agent for CMV prophylaxis and treatment in transplant populations^3^. The antiviral activity of ganciclovir requires intracellular phosphorylation and activation by the viral UL97 kinase and UL54 DNA polymerase, where ganciclovir monophosphate undergoes further phosphorylation to ganciclovir triphosphate by cellular kinases, ultimately inhibiting CMV DNA polymerase^4^. Once formed, ganciclovir triphosphate demonstrates remarkable stability with an intracellular half-life of 16.5 h, persisting in CMV-infected cells for several days and providing sustained antiviral effects^5^. However, the clinical utility of oral ganciclovir is severely limited by its poor bioavailability of approximately 6–9%, necessitating intravenous administration for therapeutic efficacy^6^. To address this limitation, valganciclovir was developed as an L-valine ester prodrug that demonstrates significantly improved oral bioavailability of approximately 60%^7^. Valganciclovir is rapidly absorbed from the gastrointestinal tract via peptide transporters PEPT1 and PEPT2, followed by extensive hydrolysis in the intestinal wall and liver to release the active moiety ganciclovir^8,9^. This rapid conversion process is typically modeled using first-order absorption kinetics with a lag time, reflecting the efficient enzymatic hydrolysis that occurs predominantly in the gastrointestinal tract^10^.

The pharmacokinetic profile of ganciclovir following valganciclovir administration exhibits substantial interindividual variability, with coefficient of variation values frequently exceeding 40% for key parameters including maximum concentration (Cmax), area under the concentration-time curve (AUC), and clearance^11,12^. Ganciclovir undergoes predominantly renal elimination, with approximately 90% of the drug excreted unchanged in urine within 24 h through glomerular filtration and active tubular secretion^13,14^. This renal dependency creates particular challenges in transplant recipients who often experience fluctuating kidney function, requiring frequent dose adjustments to maintain therapeutic efficacy while avoiding toxicity^15^. In addition, the narrow therapeutic window of ganciclovir further complicates clinical management, as subtherapeutic concentrations may lead to treatment failure and development of viral resistance through UL97 and UL54 gene mutations, while excessive exposure results in severe hematological toxicity including neutropenia, thrombocytopenia, and anemia^16,17^. These pharmacokinetic complexities, combined with the critical importance of maintaining ganciclovir concentrations within the therapeutic range, underscore the urgent need for robust, sensitive, and accessible analytical methods that can provide accurate drug quantification for optimizing patient outcomes.

Numerous analytical methodologies have been developed for ganciclovir quantification in biological matrices, with high-performance liquid chromatography (HPLC) coupled to various detection systems representing the predominant analytical approach^18,19^. These methods encompass diverse detection principles including spectrofluorimetric detection with excitation and emission wavelengths optimized for ganciclovir analysis^20,21^, UV spectrophotometry employing diode array or single-wavelength detection^22,23^, pulsed amperometric detection for electrochemical quantification^24^, and liquid chromatography-tandem mass spectrometry (LC-MS/MS) utilizing multiple reaction monitoring for enhanced selectivity^25,26^. Additionally, alternative methodologies such as capillary electrophoresis with UV detection^27^, electrochemical approaches using modified electrodes^28^, and surface-enhanced Raman spectroscopy have been explored for pharmaceutical applications^29^. However, these established analytical techniques present significant practical limitations that restrict their widespread clinical implementation. LC-MS/MS methods, while offering superior analytical performance, require expensive instrumentation with high reagent and maintenance costs, particularly due to the necessity of stable isotope internal standards that substantially increase operational expenses^30^. Furthermore, these sophisticated instruments are not consistently available in all clinical laboratories, and their complexity demands specialized technical expertise for routine operation and maintenance^31^. Electrochemical methods, despite their potential sensitivity, suffer from electrode fouling, limited selectivity in complex biological matrices, and susceptibility to interference from endogenous compounds and co-administered medications^32^. Capillary electrophoresis techniques, while offering rapid analysis, demonstrate poor reproducibility and limited robustness for routine clinical applications, particularly in terms of migration time variability and quantitative precision. Surface-enhanced Raman spectroscopy approaches require specialized substrate preparation and exhibit batch-to-batch variability that compromises analytical reliability. These collective limitations necessitate the development of alternative analytical strategies that can provide adequate sensitivity and accuracy while offering improved cost-effectiveness, operational simplicity, and enhanced reliability for routine therapeutic drug monitoring in diverse clinical settings.

Spectrofluorimetry represents an attractive analytical approach for pharmaceutical analysis, offering several inherent advantages including exceptional sensitivity through the measurement of fluorescence emission, cost-effective instrumentation that is widely available in clinical laboratories, and relatively simple operational procedures that do not require specialized technical expertise^33,34^. The technique demonstrates superior selectivity compared to conventional UV detection, as it measures both excitation and emission wavelengths, effectively reducing matrix interference and enhancing analytical specificity^35^. Additionally, spectrofluorimetric methods typically require smaller sample volumes and generate minimal chemical waste, contributing to improved environmental sustainability and reduced operational costs^36^. Two primary spectrofluorimetric approaches have been explored for ganciclovir determination: direct native fluorescence measurement and chromatographic methods coupled with fluorescence detection. Direct spectrofluorimetric determination based on ganciclovir’s native fluorescence in strongly acidic conditions (0.2 M HCl, pH 1.2) has been reported for pharmaceutical formulations, with excitation at 257 nm and emission at 374 nm achieving detection limits of 0.010 µg/mL^37^. However, this approach remains strictly limited to simple pharmaceutical matrices and cannot be extended to bioanalytical applications. The extremely harsh acidic conditions required for fluorescence enhancement cause protein denaturation and sample degradation, rendering the method incompatible with plasma or serum analysis. More critically, the UV-region spectral characteristics (λex = 257 nm, λem = 374 nm) create selectivity challenges in biological samples, where the method cannot distinguish ganciclovir fluorescence from the intense native fluorescence of endogenous compounds including aromatic amino acids (tryptophan, tyrosine, phenylalanine), plasma proteins (albumin, globulins), nucleotides, and numerous metabolites that exhibit overlapping spectral signatures.

Alternatively, existing HPLC-fluorescence methods for ganciclovir determination attempt to address these selectivity challenges through chromatographic separation but present several analytical limitations that restrict their widespread clinical implementation^20,21^. Similarly, these methods exploit the drug’s native fluorescence properties (emission at 380 nm with excitation at 260 nm) which still coincides with the fluorescence characteristics of numerous endogenous compounds and potential interferents present in biological matrices^20,21^. In addition, the methods require extensive and tedious optimization procedures to achieve adequate chromatographic separation, with studies demonstrating the necessity for precise pH adjustments from 2.60 to 2.90 in mobile phase composition to resolve critical interferences such as the aciclovir metabolite CMMG^21^. Furthermore, these approaches demand prolonged chromatographic run times exceeding 42 min, including equilibration and re-equilibration steps, which severely limits analytical throughput and increases operational costs for routine therapeutic drug monitoring^21^. These collective limitations highlight the urgent need for alternative analytical approaches that can overcome the inherent spectral and matrix interference challenges while providing improved operational efficiency and analytical robustness for clinical applications.

To address these analytical challenges, the utilization of fluorescent probes emerges as a promising alternative strategy that can significantly enhance the spectrofluorimetric determination of pharmaceuticals in complex biological matrices. Among various fluorescent probes, Erythrosin B represents a particularly attractive option due to its unique photophysical properties and analytical advantages^38^. Erythrosin B exhibits intense fluorescence in the visible region with well-separated excitation and emission wavelengths, effectively minimizing spectral overlap with endogenous fluorophores and reducing matrix interference issues commonly encountered with UV-region fluorescence detection^39^. The probe demonstrates excellent photostability under analytical conditions, ensuring consistent and reproducible fluorescence measurements throughout extended analytical sequences^40^. Furthermore, Erythrosin B shows remarkable sensitivity to its local chemical environment, enabling the development of fluorescence quenching-based analytical methods that can provide enhanced selectivity and sensitivity for target analyte determination^41^. Additionally, the use of Erythrosin B as a fluorescent probe enables the development of environmentally sustainable analytical methods with reduced solvent consumption, simplified instrumentation requirements, and elimination of derivatization steps, thereby addressing the growing demand for green analytical chemistry approaches in pharmaceutical analysis^42^.

Therefore, the primary objective of this research is to develop and validate a novel spectrofluorimetric method based on Erythrosin B fluorescence quenching for sensitive ganciclovir determination in rabbit plasma following valganciclovir administration. This represents the first reported application of Erythrosin B as a fluorescent probe for ganciclovir analysis, offering a novel alternative to existing analytical approaches including native fluorescence methods. The study will encompass comprehensive spectral characterization of Erythrosin B, ganciclovir, and their interaction complex using UV-visible absorption and fluorescence spectroscopy. The fluorescence quenching mechanism will be elucidated through Stern-Volmer kinetic analysis and temperature-dependent studies to determine molecular interaction nature. Thermodynamic parameters will be calculated to provide binding process insights, while stoichiometry will be determined using Job’s method of continuous variation. Semiempirical quantum mechanical calculations employing PM3 method will estimate binding energies and identify interaction sites. Experimental conditions affecting quenching efficiency, including pH, buffer volume, Erythrosin B concentration, and reaction time, will be systematically optimized to maximize sensitivity. The method will undergo rigorous validation according to ICH M10 bioanalytical guidelines. Subsequently, the validated method will be applied for pharmacokinetic evaluation of ganciclovir in rabbit plasma, enabling calculation of key pharmacokinetic parameters. Finally, environmental sustainability and practicality will be comprehensively assessed using different established greenness evaluation tools. This innovative approach promises to establish a sensitive, cost-effective, and environmentally sustainable analytical platform for routine therapeutic drug monitoring, addressing current analytical limitations while advancing green analytical chemistry principles in pharmaceutical analysis.

## Experimental

### Chemicals and reagents

Valganciclovir hydrochloride and ganciclovir analytical standards, Erythrosin B, and HPLC-grade acetonitrile were obtained from Sigma-Aldrich (St. Louis, MO, USA). Boric acid, phosphoric acid, acetic acid, and sodium hydroxide were purchased from Piochem Co., Cairo, Egypt. All other chemicals used were of analytical grade and were used without further purification. Distilled water was used throughout the experiments. The water quality was evaluated prior to use by measuring its fluorescence intensity under the analytical conditions (λ_ex_ = 530 nm, λ_em_ = 555 nm), which showed negligible background fluorescence, confirming no interference with the analytical measurements.

A stock solution of Erythrosin B was prepared by accurately weighing 10.0 mg of the dye and dissolving in 100 mL distilled water to obtain a concentration of 100 µg/mL (0.01% w/v). The stock solution was stored in amber-colored volumetric flasks at 4 °C in a refrigerator and protected from light to prevent photodegradation. Working solutions of different concentrations were prepared daily by appropriate dilution of the stock solution with distilled water. Britton-Robinson buffer solutions covering the pH range of 3.0–8.0 were prepared according to standard procedures. The universal buffer system was composed of 0.04 M orthophosphoric acid, 0.04 M acetic acid, and 0.04 M boric acid, with pH adjustment accomplished using 0.2 M sodium hydroxide solution. The pH of buffer solutions was verified using a calibrated pH meter before use.

### Instrumentation

Fluorescence spectroscopic measurements were performed using a Jasco FP-6200 spectrofluorometer (Jasco Inc., Easton, MD, USA) featuring a 150 W xenon light source and controlled by SpectraManager software. Operating parameters were set as follows: excitation and emission bandwidths of 10 nm, scanning velocity of 4000 nm/min, and emission detection range of 530–590 nm. UV-visible spectrophotometric analysis was carried out using a Shimadzu UV-1800 instrument (Shimadzu Corporation, Kyoto, Japan) controlled by UVProbe software with 1 cm quartz cells over the wavelength range of 200–600 nm. The spectrophotometer was operated with spectral bandwidth of 1 nm. pH determinations were made using a Jenway 3510 pH meter (Jenway Ltd., Felsted, UK) fitted with a combination glass electrode (accuracy ± 0.01 pH units). Electrode calibration was performed using certified buffer standards (pH 4.0, 7.0, and 10.0) before each measurement series. The electrode was thoroughly rinsed with distilled water between measurements to prevent cross-contamination.

### Optimization of experimental conditions

Several critical parameters affecting the fluorescence quenching efficiency were systematically optimized using a univariate approach. The effect of pH was investigated by preparing a series of Britton-Robinson buffer solutions covering the range of 3.0–8.0, and the fluorescence measurements were performed at each pH value to determine the optimal conditions for complex formation. Buffer volume optimization was conducted by varying the volume from 0.5 to 3.5 mL while maintaining constant concentrations of other components. The influence of Erythrosin B concentration was studied over the range of 2–20 µg/mL to establish the optimal probe concentration that provides maximum quenching response without interference effects. Reaction time optimization was performed by measuring the fluorescence intensity at different time intervals ranging from 1 to 8 min after mixing the reagents to ensure complete equilibrium. All optimization experiments were conducted in triplicate at room temperature, and the fluorescence intensity was measured at the emission maximum of 555 nm with excitation at 530 nm. The best condition for each parameter was selected based on maximizing the F0/F ratio, where F0 and F represent the fluorescence intensities of Erythrosin B in the absence and presence of ganciclovir, respectively.

### General analytical procedure

Into a 10 mL volumetric flask, 1.0 mL of Britton-Robinson buffer solution (pH 5.0) was added, followed by an appropriate volume of Erythrosin B stock solution to achieve a final concentration of 10 µg/mL. An appropriate volume of ganciclovir standard solution or sample solution was then added to achieve the desired final concentration within the linear range. The mixture was diluted to volume with distilled water and mixed thoroughly by gentle inversion. The resulting solution was allowed to stand for 5 min at room temperature to ensure complete complex formation and equilibrium. The fluorescence intensity was measured using the spectrofluorometer at an excitation wavelength of 530 nm and emission wavelength of 555 nm. Both excitation and emission slits were set at 10 nm bandwidth, and the photomultiplier voltage was maintained at medium sensitivity throughout all measurements.

For quantitative analysis, the fluorescence quenching was evaluated using the ratio F0/F, where F0 represents the initial fluorescence intensity of Erythrosin B alone and F represents the fluorescence intensity after addition of ganciclovir. Calibration curves were constructed by plotting F0/F values against the corresponding ganciclovir concentrations. All measurements were performed in triplicate, and the average values were used for calculations.

### Method validation

Stock solutions of ganciclovir were prepared by accurately weighing appropriate amounts of the compound and dissolving in distilled water to achieve concentrations of 100 µg/mL. These stock solutions were stored at 4 °C and protected from light. Calibration standards were prepared by serial dilution of the stock solutions using distilled water on the day of analysis at seven concentration levels: 0.05, 0.1, 0.2, 0.5, 1.0, 2.0, and 3.0 µg/mL, covering the entire analytical range in accordance with ICH M10 bioanalytical validation guidelines. Plasma sample preparation involved protein precipitation using acetonitrile. Briefly, 2 mL of acetonitrile was added to 2 mL of plasma sample, followed by vortex mixing for 2 min and centrifugation at 12,000 rpm for 10 min at 4 °C. The clear supernatant was carefully transferred and evaporated under a gentle nitrogen stream at room temperature. The dried residue was reconstituted in 1 mL of distilled water and used for spectrofluorimetric analysis under the optimized experimental conditions. The developed spectrofluorimetric method was validated according to ICH M10 bioanalytical method validation guidelines. Validation parameters assessed included linearity, accuracy, precision, selectivity, limits of detection and quantification, robustness, stability and matrix effects. Quality control (QC) samples were prepared by spiking drug-free rabbit plasma with known concentrations of ganciclovir to achieve low (0.15 µg/mL), medium (1.5 µg/mL), and high (2.25 µg/mL) concentration levels.

### Pharmacokinetic studies

A pharmacokinetic evaluation was performed using New Zealand white rabbits weighing 2.5–3.0 kg. All experimental procedures involving animals were conducted in strict accordance with the National Research Council’s Guide for the Care and Use of Laboratory Animals as well as ARRIVE (Animal Research: Reporting of In Vivo Experiments) guidelines and received ethical approval from the Institutional Ethics Committee at the Faculty of Medicine, Al-Azhar University, Damietta campus, Egypt (Approval No: DFM-IRB 00012367-25-07–039). The study utilized five rabbits that received a single oral administration of valganciclovir hydrochloride at a dose of 5 mg/kg body weight delivered through oral gavage. Serial blood sampling (4 mL per collection) was performed via the marginal ear vein according to the following schedule: 0.5, 1, 1.5, 2, 2.5, 3, 4, 6, 12, and 18 h following drug administration. Each blood sample was promptly transferred to EDTA-containing tubes and centrifuged to obtain plasma, which was subsequently frozen at −80 °C pending analysis. Plasma ganciclovir concentrations were determined using the validated spectrofluorimetric assay based on Erythrosin B fluorescence quenching. Non-compartmental pharmacokinetic analysis was conducted using PK Solver software to derive key parameters including Cmax, Tmax, AUC0-t, AUC0-∞, elimination rate constant (k), half-life (t1/2), and mean residence time (MRT).

### Mechanistic investigation and quantum mechanical calculations

The fluorescence quenching mechanism was investigated through Stern-Volmer kinetic analysis at different temperatures (298, 303, and 313 K). Fluorescence measurements were performed by systematically varying ganciclovir concentrations while maintaining constant Erythrosin B concentration. The Stern-Volmer equation (F0/F = 1 + Ksv[Q]) was applied to determine the Stern-Volmer quenching constant (Ksv), where F0 and F represent the fluorescence intensities in the absence and presence of quencher, respectively, and [Q] is the quencher concentration. Temperature-dependent fluorescence quenching studies were conducted to calculate thermodynamic parameters including enthalpy change (ΔH), entropy change (ΔS), and Gibbs free energy change (ΔG). The van’t Hoff equation (ln Ka = -ΔH/RT + ΔS/R) was utilized to determine these parameters from the temperature dependence of the association constant (Ka). The nature of intermolecular forces involved in the complex formation was elucidated based on the signs and magnitudes of the thermodynamic parameters. The stoichiometry of the Erythrosin B-ganciclovir complex was determined using Job’s method of continuous variation. Stock solutions of Erythrosin B and ganciclovir were prepared at 4.00 × 10^− 5^ M concentration. Solutions containing varying molar ratios of the two components were prepared while maintaining a constant total molar concentration of 4.00 × 10^− 6^ M. The fluorescence intensity changes were plotted against the mole fraction of ganciclovir to identify the optimal binding ratio. Semiempirical quantum mechanical calculations were performed using the PM3 method implemented in Gaussian 09 software to investigate the molecular interactions and binding mechanism. The individual geometries of Erythrosin B and ganciclovir were first optimized, followed by optimization of the complex structure. Binding energies and HOMO-LUMO energy gaps were calculated to provide insights into the interaction sites and electronic properties of the formed complex.

## Results and discussion

### Spectroscopic characterization

**Fig. 1:**
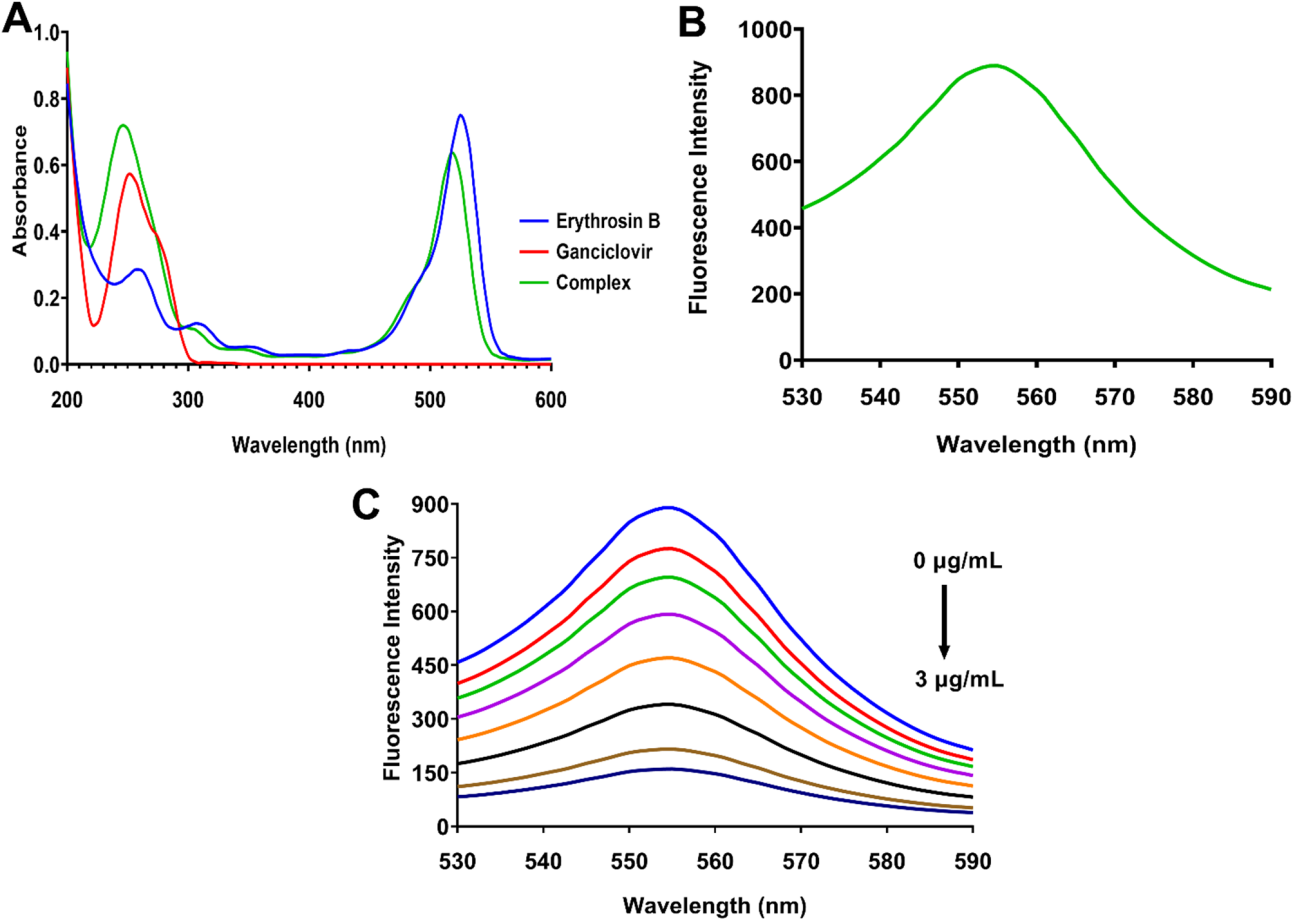
UV-visible absorption and fluorescence spectroscopic characterization of Erythrosin B-ganciclovir interaction. (**A**) UV-visible absorption spectra of Erythrosin B, ganciclovir, and their complex showing hypochromic and hypsochromic effects upon complex formation. (**B**) Fluorescence emission spectrum of Erythrosin B at λex = 530 nm showing maximum emission at 555 nm. (**C**) Fluorescence quenching of Erythrosin B upon successive addition of ganciclovir concentrations (0–3.0 µg/mL) at λex = 530 nm and λem = 555 nm.

The UV-visible absorption spectra of Erythrosin B, ganciclovir, and their complex provided essential insights into the nature of their interaction (Fig. 1A). Erythrosin B exhibited characteristic absorption bands in the visible region with a prominent peak around 530 nm, corresponding to the π-π* electronic transition of the xanthene chromophore. Ganciclovir displayed absorption in the UV region below 300 nm, attributed to the purine nucleoside structure with π-π* transitions of the guanine base, while showing no significant absorption above 300 nm. Upon complex formation between Erythrosin B and ganciclovir, significant spectral changes were observed, including a hypochromic effect characterized by decreased absorbance intensity and a hypsochromic shift toward shorter wavelengths (Fig. 1A). These spectral modifications are indicative of ground-state complex formation through intermolecular interactions between the fluorescent probe and the analyte. The hypochromic effect suggests the formation of non-fluorescent ground-state complexes, while the blue shift indicates alterations in the electronic environment of the chromophore due to molecular association. These observations confirm the establishment of a stable ground-state complex between Erythrosin B and ganciclovir, which serves as the basis for the fluorescence quenching mechanism.

The fluorescence emission spectrum of Erythrosin B demonstrated intense fluorescence with a maximum emission wavelength at 555 nm when excited at 530 nm (Fig. 1B). The fluorescence intensity of Erythrosin B showed a systematic decrease upon successive addition of ganciclovir concentrations ranging from 0 to 3 µg/mL, demonstrating the quenching phenomenon (Fig. 1C). Notably, the fluorescence quenching occurred without any significant shift in the emission wavelength maximum, maintaining the characteristic emission at 555 nm throughout the concentration range studied. This preservation of the emission maximum indicates that the electronic structure and excited-state properties of Erythrosin B remain fundamentally unchanged during the interaction with ganciclovir. The progressive decrease in fluorescence intensity with increasing ganciclovir concentration provides the analytical basis for quantitative determination, as the extent of quenching correlates directly with the analyte concentration. The maintenance of spectral shape and emission maximum confirms the stability of the fluorescent probe and demonstrates that the quenching process does not involve significant perturbation of the fluorophore’s photophysical properties, ensuring reproducible analytical measurements.

### Fluorescence quenching mechanism investigation

The comprehensive characterization of the fluorescence quenching mechanism was essential to establish the theoretical basis of the analytical method and optimize its performance. Initially, potential interference mechanisms were systematically evaluated to confirm genuine molecular interactions between Erythrosin B and ganciclovir. Förster resonance energy transfer (FRET) was excluded as a possible mechanism due to the absence of significant spectral overlap between the emission spectrum of Erythrosin B and the absorption spectrum of ganciclovir (Fig. 1A and B). Similarly, the inner filter effect was eliminated through UV-visible absorption analysis, which demonstrated that ganciclovir exhibited no measurable absorbance above 300 nm, particularly in the excitation (530 nm) and emission (555 nm) wavelength regions utilized for Erythrosin B fluorescence measurements (Fig. 1A). This absence of spectral overlap at the analytical wavelengths confirmed that the observed fluorescence intensity reduction resulted from authentic molecular interactions rather than optical artifacts. The systematic exclusion of these alternative mechanisms provided confidence that the quenching phenomenon represented a genuine interaction suitable for analytical quantification, which could occur through either static quenching involving ground-state complex formation or dynamic quenching through collisional encounters.

**Fig. 2:**
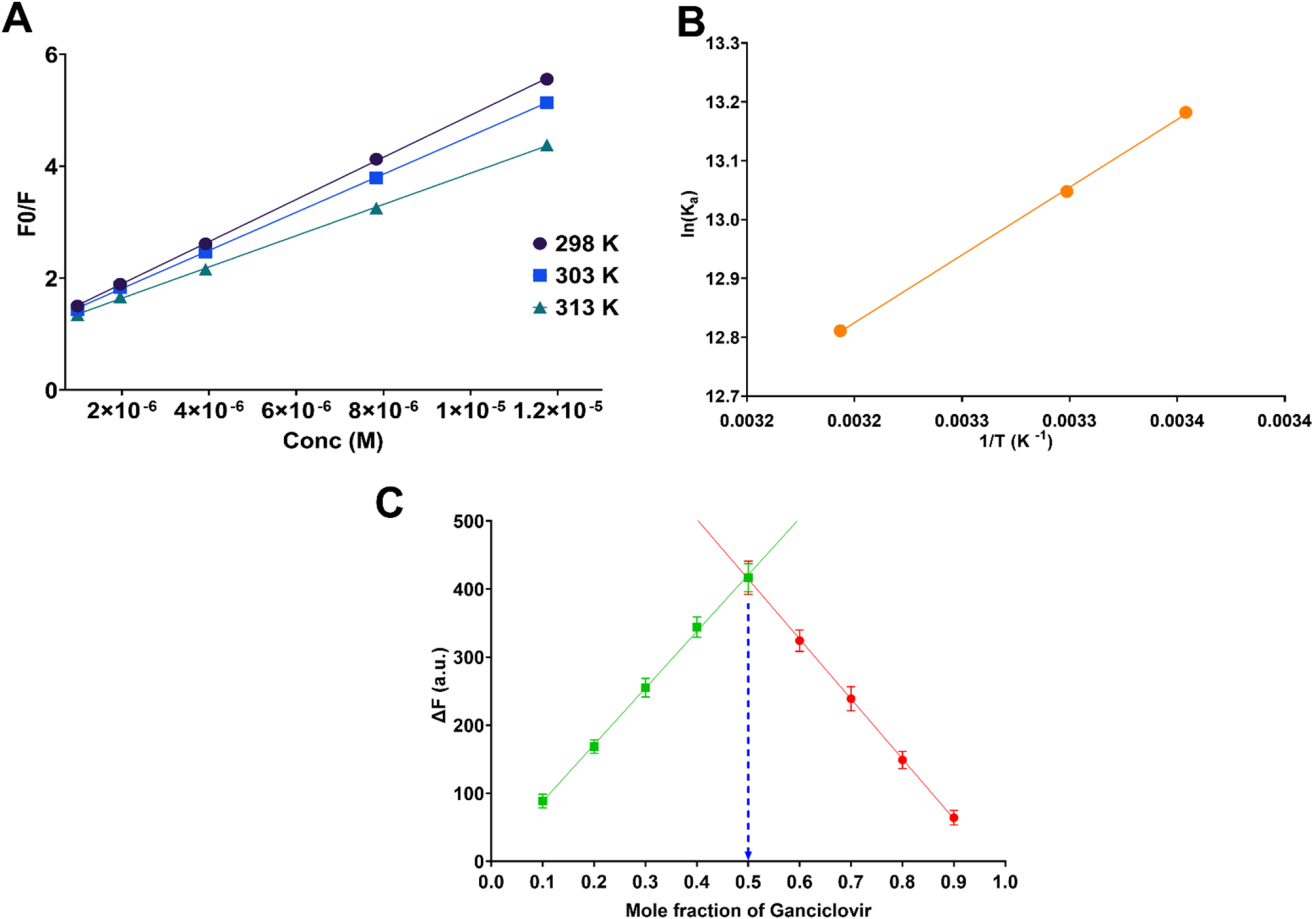
(**A**) Stern-Volmer plots at different temperatures (298, 303, and 313 K) showing linear relationships and temperature-dependent decrease in Ksv values. (**B**) van’t Hoff plot for determination of thermodynamic parameters (ΔH, ΔS, and ΔG). (**C**) Job’s method of continuous variation plot demonstrating 1:1 stoichiometry between Erythrosin B and ganciclovir with maximum at mole fraction 0.5.

Temperature-dependent Stern-Volmer analysis provided crucial insights into the nature of the quenching mechanism (Fig. 2A). Linear Stern-Volmer plots were obtained at three different temperatures (298, 303, and 313 K), yielding Stern-Volmer constants (Ksv) of 3.77 × 10^5^, 3.41 × 10^5^, and 2.79 × 10^5^ M^− 1^, respectively (Table 1). The systematic decrease in Ksv values with increasing temperature represents a characteristic signature of static quenching mechanisms, contrasting with dynamic collisional quenching where Ksv typically increases with temperature due to enhanced molecular motion and collision frequency. To further substantiate this conclusion, bimolecular quenching rate constants (Kq) were calculated using the relationship Kq = Ksv/τ₀, where τ₀ represents the fluorescence lifetime of Erythrosin B. Considering the typical fluorescence lifetime of xanthene dyes in aqueous solution (approximately 1–5 ns), the calculated Kq values ranged from 10^13^ to 10^14^ M^− 1^s^− 1^, which substantially exceed the theoretical diffusion-controlled limit of approximately 10^10^ M^− 1^s^− 1^ for dynamic quenching in aqueous media. This significant deviation from diffusion-limited kinetics provides compelling evidence for static quenching involving pre-equilibrium complex formation between Erythrosin B and ganciclovir in the ground state.

**Table 1:** Temperature-dependent stern-volmer constants (Ksv), association constants (Ka), and thermodynamic parameters for Erythrosin B-ganciclovir interaction.

Temperature (K)	Ksv (10^5^ M^− 1^)	K_a_ (10^5^ M^− 1^)	ΔG (kJ mol^− 1^)	ΔH (kJ mol^− 1^)	ΔS (J mol^− 1^ K^− 1^)
298	3.77	5.31	−32.68	−19.20	45.20
303	3.41	4.64	−32.88		
313	2.79	3.66	−33.35		

The association constants (Ka) and thermodynamic parameters provided additional mechanistic insights and quantified the strength of the molecular interaction (Table 1). Association constants (Ka) determined through modified Stern-Volmer analysis yielded values of 5.31 × 10^5^, 4.64 × 10^5^, and 3.66 × 10^5^ M^− 1^ at 298, 303, and 313 K, respectively. The substantial magnitude of these binding constants indicates strong affinity between the fluorescent probe and analyte, while their temperature-dependent decrease further corroborates the static quenching mechanism. Thermodynamic analysis using van’t Hoff plots (Fig. 2B) revealed negative Gibbs free energy values (ΔG = −32.68, −32.88, and − 33.35 kJ/mol) at the respective temperatures, confirming the spontaneous nature of complex formation. The negative enthalpy change (ΔH = −19.20 kJ/mol) indicated an exothermic interaction process primarily driven by intermolecular forces such as hydrogen bonding, electostatic interactions, and possible π-π stacking between the aromatic systems. The positive entropy change (ΔS = 45.20 J mol^− 1^ K^− 1^) suggested increased system randomness upon complex formation, likely attributed to the displacement of structured water molecules from the binding sites and conformational changes accompanying molecular association. These thermodynamic signatures collectively support a static quenching mechanism involving specific intermolecular interactions between Erythrosin B and ganciclovir.

The stoichiometry of the Erythrosin B-ganciclovir complex was definitively established through Job’s method of continuous variation (Fig. 2C). The Job’s plot demonstrated a maximum fluorescence change at a mole fraction of 0.5 for ganciclovir, unambiguously indicating the formation of a 1:1 stoichiometric complex between the fluorescent probe and analyte. This equimolar binding ratio suggests that each molecule of ganciclovir interacts with a single molecule of Erythrosin B to form the quenching complex. The symmetric bell-shaped profile of the Job’s plot and the clear maximum at the 0.5 mol fraction provide strong evidence for the formation of a well-defined, stable complex with specific stoichiometry. The establishment of 1:1 binding stoichiometry is consistent with the binding constant calculations and supports the mechanistic model of static quenching through ground-state complex formation. This stoichiometric information is crucial for understanding the molecular basis of the analytical method and ensures that the quantitative relationship between fluorescence quenching and ganciclovir concentration follows predictable thermodynamic principles, thereby enhancing the reliability and theoretical basis of the developed spectrofluorimetric assay.

### Quantum mechanical calculations

**Fig. 3:**
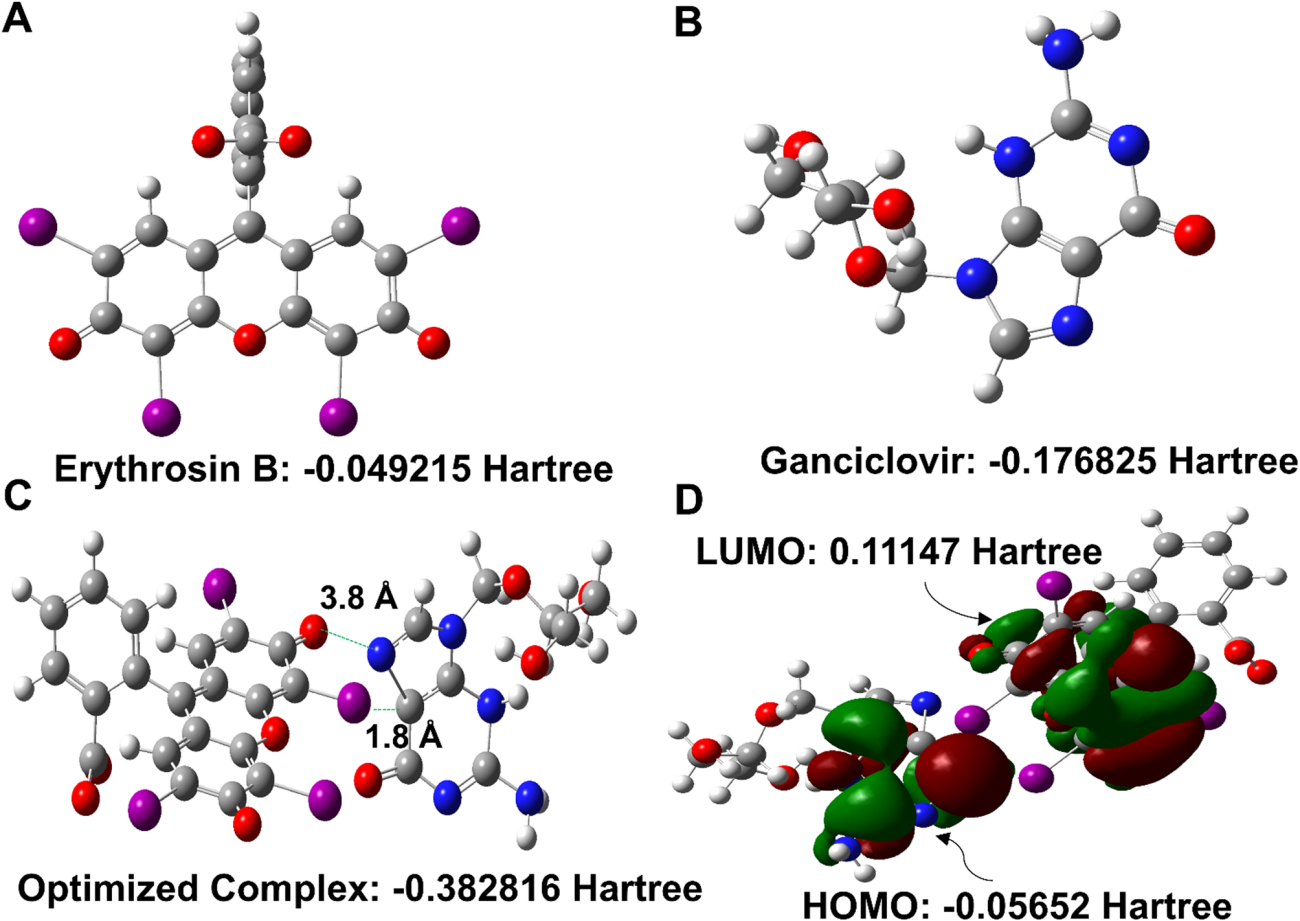
Quantum mechanical calculations using PM3 method. (**A**) Optimized geometry of Erythrosin B. (**B**) Optimized geometry of ganciclovir. (**C**) Optimized geometry of Erythrosin B-ganciclovir complex showing halogen bonding (1.8 Å) and electrostatic interactions (3.8 Å). (**D**) HOMO-LUMO energy diagram illustrating photoinduced electron transfer mechanism within the complex.

Semiempirical quantum mechanical calculations using the PM3 method were employed to determine the binding sites, elucidate the molecular interactions, and gain deeper insights into the electronic properties governing the fluorescence quenching mechanism. The PM3 method was selected for its computational efficiency and reasonable accuracy for large molecular systems, allowing for rapid optimization of the complex geometries while maintaining acceptable precision for binding energy calculations. Although density functional theory (DFT) methods would provide more accurate results, they require substantially higher computational resources and longer calculation times for systems of this size. The PM3 approach offers a favorable balance between computational feasibility and chemical accuracy for supramolecular complex analysis and provides valuable insights into the binding sites and electronic properties of the Erythrosin B-ganciclovir interaction. The optimized geometries of the individual components and their complex were determined to understand the structural basis of the fluorescence quenching phenomenon (Fig. 3). The calculated total energies for the isolated molecules were − 0.049215 Hartree for Erythrosin B (Fig. 3A) and − 0.176825 Hartree for ganciclovir (Fig. 3B), while the optimized complex exhibited a total energy of −0.382816 Hartree (Fig. 3C). The binding energy was calculated using the supramolecular approach according to the equation:

ΔE = E(complex) - E(Erythrosin B) - E(ganciclovir).

yielding a value of −0.156776 Hartree. This negative binding energy indicates a thermodynamically favorable interaction that supports the experimental observations of stable ground-state complex formation and validates the static quenching mechanism established through experimental investigations.

The analysis of specific interaction sites revealed multiple stabilizing forces contributing to complex formation. The optimized complex geometry demonstrated two primary interaction modes: halogen bonding and electrostatic interactions. A halogen bonding interaction was identified between an iodine atom of Erythrosin B and a carbon atom in the imidazole ring of the purine system of ganciclovir, with an intermolecular distance of 1.8 Å (Fig. 3C). This relatively short distance is characteristic of strong halogen bonds, where the iodine atom acts as an electron-accepting σ-hole that interacts favorably with the electron-rich π-system of the imidazole ring. Halogen bonding represents a significant non-covalent interaction that contributes substantially to molecular recognition and complex stability in supramolecular systems. Additionally, an electrostatic interaction was observed between the carbonyl oxygen of Erythrosin B and nitrogen atoms in the purine ring system of ganciclovir, with an intermolecular distance of 3.8 Å. This longer-range electrostatic interaction provides additional stabilization through charge-dipole interactions. The combination of these multiple interaction modes explains the substantial binding affinity observed experimentally and provides a molecular-level rationalization for the selective recognition between Erythrosin B and ganciclovir.

The HOMO-LUMO analysis provided crucial insights into the electronic properties and photoinduced electron transfer (PET) mechanism underlying the fluorescence quenching process (Fig. 3D). The HOMO energy of the optimized complex was calculated as −0.05652 Hartree, while the LUMO energy was determined to be 0.11147 Hartree, resulting in a HOMO-LUMO energy gap of 0.16799 Hartree. The molecular orbital distributions revealed that the HOMO is primarily localized on the ganciclovir moiety, particularly concentrated on the purine ring system, while the LUMO is predominantly distributed across the Erythrosin B structure. This spatial separation of frontier molecular orbitals creates an ideal electronic configuration for PET processes within the static quenching mechanism. The fluorescence quenching occurs through the following sequential steps:

(1) Erythrosin B + Ganciclovir ⇌ [Erythrosin B -Ganciclovir].

(2) [Erythrosin B -Ganciclovir] + hν → [Erythrosin B ^*^-Ganciclovir].

(3) [Erythrosin B ^*^-Ganciclovir] → [Erythrosin B ⁻-Ganciclovir⁺] → non-radiative deactivation.

The favorable orbital alignment between the ganciclovir HOMO (electron donor) and the Erythrosin B LUMO (electron acceptor) facilitates efficient electron transfer within the excited-state complex, leading to charge separation and subsequent non-radiative decay pathways that quench the fluorescence. The quantum mechanical calculations collectively validate the experimental findings and provide a comprehensive theoretical framework demonstrating that the fluorescence quenching occurs through a PET mechanism operating within a stable ground-state complex, thereby supporting the static quenching model and the analytical method’s molecular basis.

### Optimization of experimental conditions

**Fig. 4:**
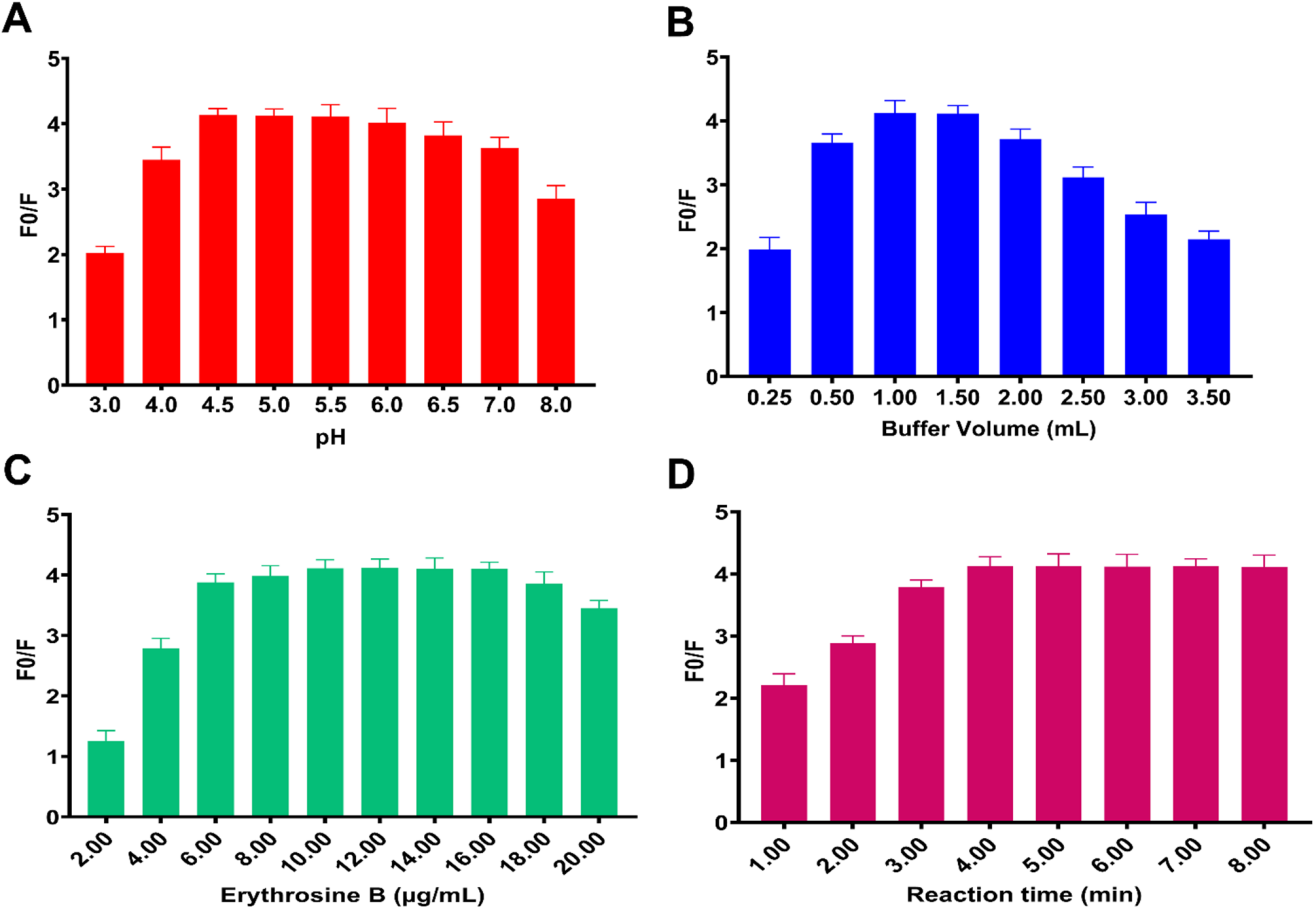
Optimization of experimental conditions presented as bar charts showing processed fluorescence quenching efficiency (F0/F). **(A)** Effect of pH on quenching efficiency showing optimum at pH 5.0; **(B)** Effect of buffer volume on quenching efficiency with optimum at 1.0 mL; **(C)** Effect of Erythrosin B concentration showing optimum at 10 µg/mL; **(D)** Effect of reaction time demonstrating rapid equilibration within 5 min. F0 represents the fluorescence intensity of Erythrosin B alone, and F represents the fluorescence intensity in the presence of 2.0 µg/mL ganciclovir. All measurements performed in triplicate at room temperature with error bars representing standard deviation (*n* = 3).

The systematic optimization of experimental conditions was essential to maximize the fluorescence quenching efficiency and ensure reproducible analytical performance. A univariate approach was employed to investigate the effects of pH, buffer volume, Erythrosin B concentration, and reaction time on the F0/F ratio, where F0 and F represent the fluorescence intensities of Erythrosin B in the absence and presence of ganciclovir, respectively. The optimization studies were conducted using 2.0 µg/mL ganciclovir as the test concentration, with all measurements performed in triplicate at room temperature. The pH optimization study revealed that the fluorescence quenching efficiency increased progressively from pH 3.0 to pH 5.0, reaching maximum efficiency at pH 5.0 (Fig. 4A). Beyond pH 5.0, the quenching efficiency gradually decreased, with notable reduction observed at pH values exceeding 6.0. This pH dependency can be attributed to the ionization states of both Erythrosin B and ganciclovir, which significantly influence their molecular interaction and complex formation ability.

Buffer volume optimization demonstrated that the F0/F ratio increased substantially from 0.5 to 1.5 mL buffer volume, reaching an optimal value at 1.0 mL (Fig. 4B). Further increases in buffer volume beyond 1.5 mL resulted in progressive decrease in quenching efficiency. At buffer volumes exceeding 1.5 mL, the increased ionic strength destabilizes the electrostatic interactions between Erythrosin B and ganciclovir, thereby reducing the complex formation efficiency and analytical sensitivity. The Erythrosin B concentration study showed rapid increase in F0/F ratio up to 10 µg/mL, achieving maximum quenching efficiency, followed by gradual decline at higher concentrations (Fig. 4C). This behavior suggests that optimal probe concentration provides sufficient binding sites without causing self-quenching or aggregation phenomena that could interfere with the analytical response. Reaction time optimization indicated that the fluorescence quenching reached equilibrium rapidly, with maximum efficiency achieved within 5 min and maintained consistently thereafter (Fig. 4D). This rapid equilibration confirms the efficient formation of the Erythrosin B-ganciclovir complex and supports the static quenching mechanism established in the mechanistic investigations. Based on these optimization studies, the following conditions were selected for subsequent analytical applications: pH 5.0 Britton-Robinson buffer (1.0 mL), Erythrosin B concentration of 10 µg/mL, and reaction time of 5 min.

### Method validation

The developed spectrofluorimetric method was comprehensively validated according to ICH M10 bioanalytical method validation guidelines^43^. Linearity was established over the concentration range of 0.05–3.0 µg/mL with excellent correlation coefficient (r^2^ = 0.9991) and the regression equation: F0/F = 1.4373 C + 1.0749, where C represents the ganciclovir concentration in µg/mL (Table 2). The limits of detection (LOD) and quantification (LOQ) were determined as 0.0157 and 0.0472 µg/mL, respectively, providing adequate sensitivity for therapeutic monitoring applications. Furthermore, accuracy and precision studies were conducted at four concentration levels including the LLOQ (0.05 µg/mL) and three additional quality control levels (0.15, 1.5, and 2.25 µg/mL). Intraday accuracy ranged from 98.87% to 104.76% with precision values below 3.45% RSD, while interday accuracy ranged from 96.04% to 103.19% with precision below 4.61% RSD across all tested concentrations (Table S1). The inter-day precision was calculated using all data from five independent analytical batches performed over three consecutive days to properly reflect between-day variability in accordance with ICH M10 guidelines. These results confirm that the method meets the stringent accuracy and precision requirements for bioanalytical applications, with all values well within the acceptable limits of ± 15% for quality control samples and ± 20% for LLOQ.

**Table 2:** Analytical performance parameters of the developed spectrofluorimetric method including linearity range, regression equation, correlation coefficient, and limits of detection and quantification.

Parameter	Ganciclovir
Linearity range (µg/mL)	0.05–3.0.05.0
Intercept (a)	1.0749
Slope (b)	1.4373
Coefficient of determination (r^2^)	0.9991
SE of intercept (Sa)	0.0269
SE of slope (Sb)	0.0188
LOD (µg/mL)	0.0157
LOQ (µg/mL)	0.0472
LLOQ (µg/mL)	0.05
ULOQ (µg/mL)	3.0

In addition, selectivity studies demonstrated the method’s high ability to distinguish ganciclovir from potential interferents, achieving approximately 75% quenching efficiency for ganciclovir while showing minimal interference from various endogenous compounds and biological components including Na⁺, K⁺, Ca²⁺, Mg²⁺, SO₄²⁻, PO₄³⁻, tryptophan, tyrosine, glutamic acid, glucose, and pooled plasma (Fig. S1). The interference from these substances remained below 3% quenching efficiency, confirming the method’s high selectivity for ganciclovir determination in complex biological matrices. Moreover, robustness evaluation showed that slight modifications in critical parameters including pH (± 0.2 units), buffer volume (± 0.2 mL), Erythrosin B concentration (± 1 µg/mL), and reaction time (± 0.2 min) had minimal impact on analytical performance, with recovery values remaining within 98.45–101.91.45.91% (Table S2). Additionally, matrix effect studies using three independent rabbit plasma sources demonstrated consistent recovery percentages between 96.21% and 104.43% across all quality control levels with coefficient of variation values below 4.2% (Table S3). The low inter-source variability observed is consistent with the controlled nature of laboratory animal studies, where standardized housing, diet, and genetic background minimize biological variability compared to human populations. While ICH M10 guidelines recommend at least 6 independent matrix sources, the consistent results across three quality control levels and three independent plasma lots provide reasonable assurance of method reliability for this preclinical animal model, confirming minimal matrix interference for biological matrix analysis. Comprehensive stability studies demonstrated that ganciclovir in rabbit plasma remained stable under all tested storage and handling conditions (Table S4). Refrigerated samples (2–8 °C, 24 h) and processed samples in the autosampler (10 °C, 48 h) exhibited excellent stability with recoveries of 98.65–100.45.65.45%. Short-term room temperature stability (24 h) showed acceptable recoveries of 96.23–97.18%. Freeze-thaw stability through three cycles and long-term storage at −80 °C for 30 days demonstrated recoveries of 92.15–95.34%, indicating that repeated freeze-thaw cycles and extended frozen storage should be minimized when possible though all stability conditions met the 85–115% acceptance criteria with CV values below 5.0%, confirming the method’s reliability under routine laboratory conditions in accordance with ICH M10 guidelines. These comprehensive validation results demonstrate that the developed method meets the stringent requirements for bioanalytical method validation and is suitable for routine therapeutic drug monitoring applications.

### Pharmacokinetic studies

**Fig. 5:**
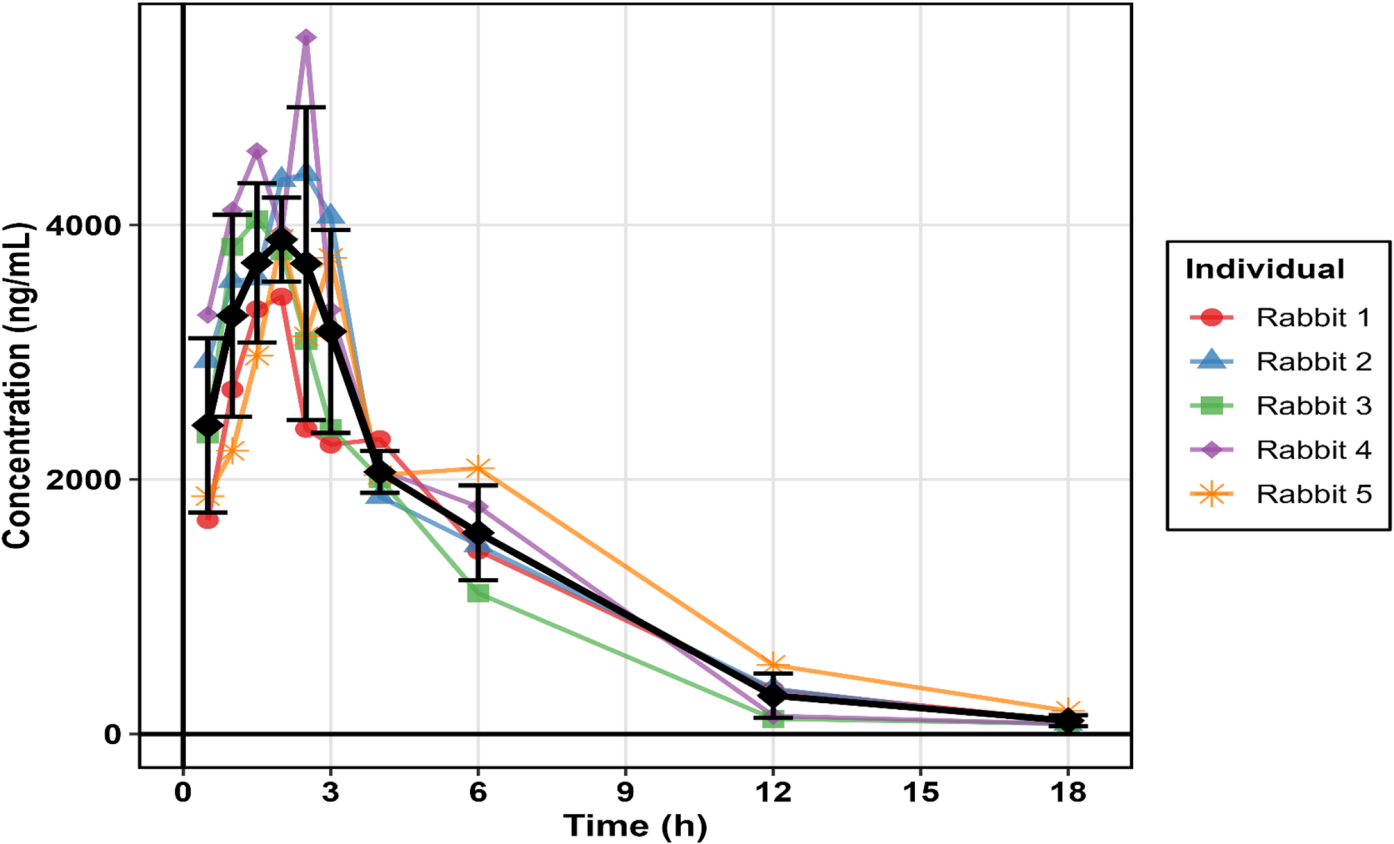
Plasma concentration-time profile of ganciclovir in New Zealand white rabbits (*n* = 5) following oral administration of valganciclovir hydrochloride (5 mg/kg). Individual rabbit profiles are shown with distinct colors and markers (red circles, blue triangles, green squares, purple diamonds, orange asterisks), while the mean concentration profile is displayed as a bold black line with diamond markers. Error bars represent standard deviation.

The validated spectrofluorimetric method was successfully applied for pharmacokinetic evaluation of ganciclovir in New Zealand white rabbits following oral administration of valganciclovir hydrochloride at 5 mg/kg body weight. The concentration-time profile demonstrated characteristic pharmacokinetic behavior consistent with previously reported studies (Fig. 5). A comprehensive analysis of the pharmacokinetic parameters was conducted using non-compartmental analysis, providing valuable insights into the absorption, distribution, and elimination characteristics of ganciclovir in this experimental model. The study represents a significant advancement in analytical methodology, as it demonstrates the first successful application of an Erythrosin B-based spectrofluorimetric method for pharmacokinetic analysis of ganciclovir, offering a cost-effective and environmentally sustainable alternative to conventional LC-MS/MS approaches.

#### Absorption and distribution parameters

The absorption phase was characterized by rapid uptake following valganciclovir administration, with maximum plasma concentrations (Cmax) of 4.25 ± 0.77 µg/mL achieved at a median time to maximum concentration (Tmax) of 2.0 h (range 1.5–2.5 h) (Table 3). These findings align closely with published clinical data, where valganciclovir demonstrates rapid absorption with Tmax values typically ranging from 1.0 to 3.5 h in transplant populations^6^. The observed Tmax values confirm the efficient conversion of valganciclovir to ganciclovir through enzymatic hydrolysis mediated by intestinal and hepatic esterases, as described in population pharmacokinetic models that incorporate first-order absorption kinetics with lag time parameters^3^. The coefficient of variation for Tmax (19.9%) indicates moderate inter-animal variability, which is consistent with clinical observations where absorption parameters show greater variability compared to elimination parameters due to factors affecting gastrointestinal absorption and first-pass metabolism^9^.

**Table 3:** Pharmacokinetic parameters of ganciclovir in new Zealand white rabbits following oral administration of valganciclovir hydrochloride (5 mg/kg).

Pharmacokinetic Parameter	Value	CV (%)
Absorption		
C_max_ (µg/mL)	4.25 ± 0.77	18.1
T_max_ (h)ᵃ	2.0 (1.5–2.5)	19.9
Distribution		
Vz/F (L)	0.99 ± 0.13	12.9
Elimination		
t½ (h)	2.94 ± 0.32	11.0
CL/F (L/h)	0.24 ± 0.03	13.2
Exposure		
AUC_0→ t_ (µg·h/mL)	21.1 ± 2.6	12.3
AUC_0→ ∞_ (µg·h/mL)	21.6 ± 2.7	12.6
MRT (h)	4.52 ± 0.71	15.6

ᵃ Median (range)

The apparent volume of distribution (Vz/F) was determined to be 0.99 ± 0.13 L with a low coefficient of variation of 12.9% (Table 3), indicating consistent distribution characteristics across the study population. This value is remarkably consistent with clinical reports where ganciclovir demonstrates limited tissue distribution, primarily to extracellular fluid compartments. Czock et al. reported a central volume of distribution (Vc) of 0.213 L/kg in HIV-positive and CMV-positive patients^44^, while population pharmacokinetic studies have documented central volumes ranging from 5.2 to 87.5 L in various clinical populations when administered as valganciclovir^45,46^. The limited volume of distribution observed reflects ganciclovir’s hydrophilic nature and minimal plasma protein binding (1–2%), which restricts its distribution primarily to extracellular fluid spaces^13^. This distribution pattern is clinically relevant as it ensures adequate drug concentrations in plasma and extracellular compartments where viral replication occurs, while minimizing unnecessary tissue accumulation that could contribute to toxicity.

#### Elimination and clearance characteristics

The elimination phase revealed a half-life (t½) of 2.94 ± 0.32 h and total clearance (CL/F) of 0.24 ± 0.03 L/h, both demonstrating excellent precision with coefficient of variation values below 13.2% (Table 3). These elimination parameters are consistent with the known pharmacokinetic properties of ganciclovir, which undergoes predominantly renal elimination through glomerular filtration and active tubular secretion. The observed half-life values in this rabbit model fall within the range reported in clinical studies, where ganciclovir half-life varies from 2.5 to 6.0 h depending on renal function and patient population^47^.

The total clearance value of 0.24 ± 0.03 L/h observed in this study reflects the efficient renal elimination of ganciclovir, which is consistent with the drug’s elimination pathway where approximately 90% is excreted unchanged in urine within 24 h^13^. Population pharmacokinetic studies have demonstrated significant correlations between ganciclovir clearance and creatinine clearance, with clearance values ranging from 0.6 L/h in renal transplant patients to 24.2 L/h in healthy volunteers, emphasizing the critical role of renal function in drug elimination^2^. The area under the concentration-time curve (AUC_0−∞_) of 21.6 ± 2.7 µg·h/mL and mean residence time (MRT) of 4.52 ± 0.71 h provide important exposure information that can be extrapolated to clinical relevance. The exposure levels achieved in this rabbit model suggest adequate systemic availability for therapeutic efficacy, particularly considering the single-dose administration protocol employed in this study. The pharmacokinetic parameters obtained using the developed spectrofluorimetric method demonstrate remarkable consistency with published clinical data, validating both the analytical methodology and the experimental model. These findings collectively establish the developed method as a reliable and practical tool for pharmacokinetic studies and therapeutic drug monitoring of ganciclovir in preclinical and clinical settings.

### Green assessment and environmental sustainability

**Fig. 6:**
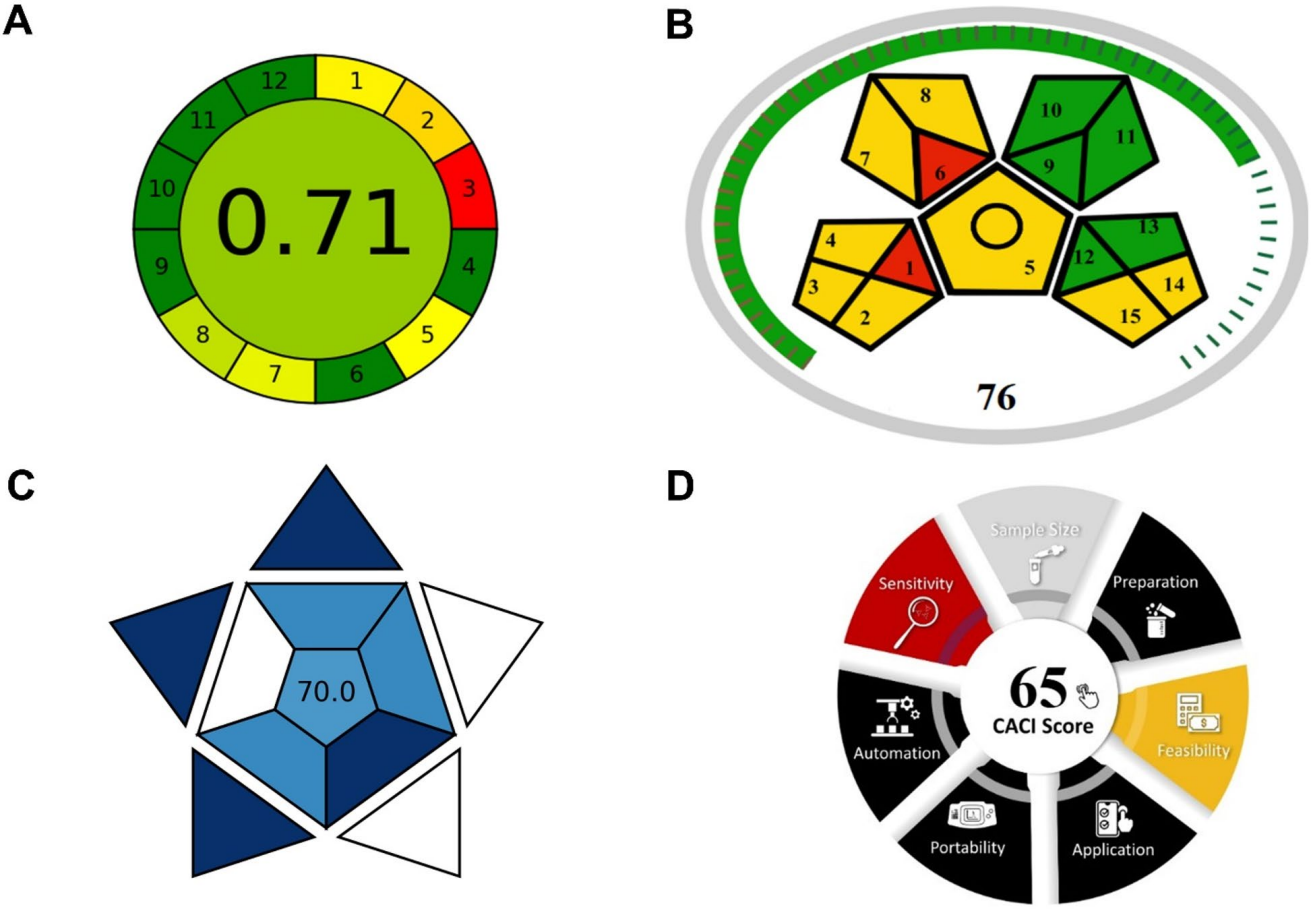
Green assessment evaluation of the developed spectrofluorimetric method using four assessment tools: (**A**) AGREE assessment (score: 0.71) based on 12 SIGNIFICANCE principles with green, yellow, and red segments indicating excellent, moderate, and poor environmental performance, respectively; (**B**) MoGAPI evaluation (score: 76%) showing green analytical procedure characteristics; (**C**) BAGI assessment (score: 70.0) demonstrating practical applicability with dark blue, light blue, and white segments representing excellent, moderate, and poor practicality, respectively; (**D**) CACI evaluation (score: 65%) indicating acceptable click analytical chemistry performance with colored, gray, and black segments showing good, moderate, and poor operational characteristics, respectively.

The environmental sustainability and practical applicability of the developed spectrofluorimetric method were comprehensively evaluated using four established assessment tools, with Analytical GREEnness (AGREE)^48^ and Modified Green Analytical Procedure Index (MoGAPI)^49^ focusing on green analytical chemistry principles, while Blue Applicability Grade Index (BAGI)^50^ and Click Analytical Chemistry Index (CACI)^51^ evaluating analytical practicality and operational efficiency (Fig. 6). This multi-dimensional assessment approach provides a comprehensive evaluation framework that addresses both environmental impact and practical implementation considerations, responding to the increasing demand for sustainable analytical methodologies in pharmaceutical analysis.

The AGREE assessment revealed an overall score of 0.71, classifying the method as environmentally acceptable according to the 12 SIGNIFICANCE principles of green analytical chemistry (Fig. 6A). The method demonstrated excellent environmental performance in several critical areas, particularly achieving maximum scores for derivatization avoidance (Principle 6), as the spectrofluorimetric approach eliminates the need for chemical derivatization steps commonly required in conventional analytical methods. The energy consumption criterion (Principle 9) scored favorably due to the use of spectrofluorimetric instrumentation, which typically consumes less than 0.1 kWh per sample compared to energy-intensive techniques such as liquid chromatography-tandem mass spectrometry (LC-MS/MS) systems that require higher kWh per analysis. The method’s moderate performance in sample size requirements (Principle 2) reflects the use of 2 mL plasma samples, which, while reasonable for bioanalytical applications, represents a compromise between analytical sensitivity and sample conservation.

The MoGAPI evaluation yielded a score of 76%, demonstrating excellent green analytical procedure characteristics (Fig. 6B). This assessment particularly highlighted the method’s environmental strengths in reagent selection and waste minimization strategies. The exclusive use of commercially available, low-toxicity reagents including Erythrosin B (10 µg/mL final concentration), acetonitrile for protein precipitation (2 mL per sample), and standard buffer components significantly contributed to the favorable environmental profile. Waste generation assessment revealed minimal environmental impact, with total waste production limited to approximately 4 mL per sample, consisting primarily of acetonitrile used for protein precipitation and aqueous buffer solutions that can be safely disposed through standard laboratory waste management protocols. The method’s excellent performance in health and safety criteria stems from the elimination of highly toxic solvents and the use of reagents with favorable National Fire Protection Association (NFPA) ratings, with Erythrosin B classified as minimally hazardous and acetonitrile representing the only organic solvent with well-established safety protocols.

The BAGI assessment score of 70.0 confirmed the method’s practical applicability for routine bioanalytical applications (Fig. 6C). The evaluation emphasized the method’s quantitative analytical capabilities, enabling accurate determination of ganciclovir concentrations across the therapeutic range of 0.05–3.0 µg/mL with excellent precision and accuracy meeting ICH M10 bioanalytical validation requirements. The instrumental requirements score favorably due to the widespread availability of spectrofluorometers in analytical laboratories, contrasting with specialized LC-MS/MS systems that require substantial capital investment and technical expertise. Sample throughput analysis demonstrated the method’s efficiency over conventional high-performance liquid chromatography (HPLC) methods requiring 42-minute run times. The reagent accessibility also scored excellently due to the commercial availability of all required materials without need for specialized synthesis or procurement procedures.

The CACI evaluation provided a score of 65%, indicating acceptable performance within the click analytical chemistry framework focusing on operational simplicity and efficiency (Fig. 6D). The assessment highlighted the method’s cost-effectiveness, with reagent costs estimated at less than $2 per sample analysis, primarily attributed to the minimal quantities of Erythrosin B required (10 µg/mL) and standard laboratory consumables. Feasibility scored excellently due to the exclusive use of commercially available reagents and standard analytical instrumentation accessible in most analytical laboratories. The method demonstrated good quantitative analytical capability with single-analyte determination suitable for therapeutic drug monitoring applications. Sample preparation requirements scored moderately, reflecting the necessary protein precipitation step using acetonitrile, while analysis time achieved favorable scoring with complete determination possible within 5 min of fluorescence measurement.

Despite the overall favorable assessment results, few limitations were identified that present opportunities for future methodological improvements. The primary limitation across all assessment tools was the requirement for off-line laboratory-based analysis, restricting point-of-care applications and real-time monitoring capabilities. The single-analyte nature of the method limits its efficiency for multi-drug therapeutic monitoring scenarios common in transplant populations receiving complex medication regimens. Sample preparation requirements, while minimal compared to conventional methods, still necessitate protein precipitation and centrifugation steps that could be further streamlined. Future improvements could focus on developing flow injection analysis or automated sample handling systems to enhance throughput and reduce manual intervention. Implementation of portable or miniaturized fluorescence detection systems could address portability limitations and enable near-patient testing applications. Additionally, expanding the methodology to simultaneous multi-analyte determination through spectral deconvolution or chemometric approaches would significantly enhance practical utility. The development of alternative sample preparation strategies, such as direct fluorescence measurement in diluted plasma or implementation of online sample clean-up procedures, could further improve the environmental and practical profile while maintaining analytical performance standards.

## Conclusions

This study successfully developed and validated a novel spectrofluorimetric method based on Erythrosin B fluorescence quenching for sensitive determination of ganciclovir in rabbit plasma following valganciclovir administration. The comprehensive analytical approach represents a significant advancement in green analytical chemistry applications for pharmaceutical analysis, offering a cost-effective and environmentally sustainable alternative to conventional LC-MS/MS methodologies. The method’s foundation on static quenching mechanism was rigorously established through temperature-dependent Stern-Volmer analysis, revealing Stern-Volmer constants ranging from 3.77 × 10⁵ to 2.79 × 10⁵ M⁻¹ across the temperature range studied. Furthermore, thermodynamic analysis provided crucial insights into the interaction nature, with negative Gibbs free energy values confirming spontaneous complex formation, while the calculated binding energies from semiempirical PM3 quantum mechanical calculations validated the experimental observations. The 1:1 stoichiometry established through Job’s method, combined with the identification of halogen bonding and electrostatic interactions as primary binding mechanisms, provided a comprehensive molecular-level understanding of the fluorescence quenching process.

The analytical performance characteristics demonstrated excellent suitability for bioanalytical applications, with the method achieving good linearity (r² = 0.9991) across the therapeutic concentration range of 0.05–3.0 µg/mL and adequate sensitivity reflected in limits of detection and quantification of 0.0157 and 0.0472 µg/mL, respectively. Moreover, comprehensive validation according to ICH M10 bioanalytical guidelines confirmed the method’s reliability, with accuracy values ranging from 96.04% to 104.76% and precision below 4.61% RSD across all quality control levels, meeting stringent regulatory requirements for therapeutic drug monitoring applications. The successful pharmacokinetic application in New Zealand white rabbits yielded key parameters including Cmax of 4.25 ± 0.77 µg/mL, half-life of 2.94 ± 0.32 h, and AUC₀₋∞ of 21.6 ± 2.7 µg·h/mL, which demonstrated remarkable consistency with published clinical data, thereby validating both the analytical methodology and the experimental model. Additionally, the environmental sustainability assessment using four complementary evaluation tools (AGREE: 0.71, MoGAPI: 76%, BAGI: 70.0, CACI: 65%) confirmed the method’s favorable green analytical chemistry credentials, demonstrating superior environmental performance compared to conventional analytical approaches through reduced waste generation, minimal energy consumption, and elimination of derivatization requirements.

### Challenges and future perspectives

Despite the significant achievements in the developed method, several limitations warrant acknowledgment and present opportunities for future methodological enhancements. The primary limitation involves the lack of cross-validation with established LC-MS/MS reference methods, which would strengthen the method’s credibility and facilitate broader acceptance in clinical laboratories. Additionally, selectivity challenges may arise from the fact that numerous compounds have been reported as potential quenchers for Erythrosin B, potentially compromising analytical specificity in complex biological matrices containing multiple fluorescence-interfering substances. However, these selectivity limitations could be effectively addressed through implementation of selective sample preparation protocols, such as molecularly imprinted polymers (MIPs) specifically designed for ganciclovir recognition, which would enhance selectivity while maintaining the method’s green analytical chemistry advantages. Furthermore, the current off-line laboratory-based approach restricts point-of-care applications and real-time monitoring capabilities, limiting its utility in emergency clinical situations requiring immediate therapeutic decisions.

Future research directions should focus on developing automated flow injection analysis systems to enhance throughput and reduce manual intervention, while implementation of portable or miniaturized fluorescence detection systems could address portability limitations and enable near-patient testing applications. Moreover, expanding the methodology to simultaneous multi-analyte determination through advanced chemometric approaches or spectral deconvolution techniques would significantly enhance practical utility for complex therapeutic monitoring scenarios common in transplant populations. The integration of artificial intelligence and machine learning algorithms for spectral interpretation and interference correction represents another promising avenue for method enhancement. Additionally, comprehensive cross-validation studies with established LC-MS/MS methods across diverse patient populations would strengthen the method’s clinical relevance and facilitate regulatory acceptance. Finally, the development of miniaturized sample preparation devices incorporating molecularly imprinted polymers or other selective extraction media could further improve the environmental profile while addressing selectivity concerns, ultimately establishing this spectrofluorimetric approach as a viable alternative for routine ganciclovir therapeutic monitoring in both preclinical and clinical settings.

## Supplementary Information

Below is the link to the electronic supplementary material.

Supplementary Material 1

## Data Availability

The data presented in this study are available on request from the corresponding author.
