# Supplementary material for: Validated Erythrosin B spectrofluorimetric method for ganciclovir bioanalysis in rabbit plasma following valganciclovir bioconversion and Pharmacokinetic application
Source: Sci Rep. 2025 Dec 23;15:44415. doi: 10.1038/s41598-025-32405-z (PMC12739150; doi:10.1038/s41598-025-32405-z)
Supplement: Supplementary file 1 — Supplementary Material 1 [file 41598_2025_32405_MOESM1_ESM.docx]

**Validated Erythrosin B Spectrofluorimetric Method for Ganciclovir Bioanalysis in Rabbit Plasma Following Valganciclovir Bioconversion and Pharmacokinetic Application**

**Ali Alqahtani ^a^,** **Taha Alqahtani ^a^,** **Adil Alshehri ^b^,** **Ahmed A. Almrasy ^c, *^**

^a^ Department of Pharmacology, College of Pharmacy, King Khalid University, Abha, 62529, Saudi Arabia

^b^ Department of Medicine, College of Medicine, King Khalid University, Abha, 62529, Saudi Arabia

^c^ Pharmaceutical Analytical Chemistry Department, Faculty of Pharmacy, Al-Azhar University, Cairo 11751, Egypt

*Corresponding author email address) **Ahmed A. Almrasy**): [ahmedalialmrasy8@gmail.com](mailto:ahmedalialmrasy8@gmail.com)

**Table S1:** Intraday and interday accuracy and precision data for the spectrofluorimetric determination of ganciclovir at four concentration levels.

| *Concentration (µg/mL)* | | *Intraday* [*^a^*](https://www.sciencedirect.com/science/article/pii/S1386142524003305#tblfn1) | | *Interday* ^b^ | |
| --- | --- | --- | --- | --- | --- |
|  |  | *Accuracy*  *(% R ± SD)* | *Precision (*RSD%*)* | *Accuracy*  *(% R ± SD)* | *Precision (*RSD%*)* |
| 0.05 | 102.23 ± 3.525 | | 3.448 | 96.04 ± 3.295 | 4.612 |
| 0.15 | 104.76 ± 2.677 | | 2.556 | 96.19 ± 3.627 | 3.771 |
| 1.50 | 103.44 ± 1.776 | | 1.717 | 103.19 ± 2.275 | 2.204 |
| 2.25 | 98.87 ± 1.826 | | 1.847 | 98.97 ± 2.59 | 2.616 |

^a^ Intra-day: Average of five determinations within a single analytical batch.

^b^ Inter-day: Calculated from all data obtained from five independent analytical batches performed over three consecutive days.

**Table S2:** Robustness evaluation showing the effect of slight modifications in critical parameters on analytical performance.

| Parameter | Modification | % Recovery ± SD |
| --- | --- | --- |
|  | 4.8 | 101.42 ± 1.31 |
| Buffer (pH) | 5.0 (optimum) | 98.69 ± 0.838 |
|  | 5.2 | 100.25 ± 0.71 |
|  | 9 | 98.45 ± 1.517 |
| Erythrosine B Conc. (µg/mL) | 10 (optimum) | 101.12 ± 1.263 |
|  | 11 | 98.14 ± 1.624 |
|  | 4.8 | 101.18 ± 1.484 |
| Incubation time (min) | 5 (optimum) | 101.91 ± 1.206 |
|  | 5.2 | 98.78 ± 0.768 |

**Table S3:** Matrix effect studies using different batches of rabbit plasma showing recovery percentages, coefficient of variation (CV%), and bias values at three quality control levels.

|  | Plasma 1 | | | | Plasma 2 | | | | Plasma 3 | | |
| --- | --- | --- | --- | --- | --- | --- | --- | --- | --- | --- | --- |
| Concentration (µg/mL) | Recovery % | (CV%) | (%Bias) | Recovery % | | (CV%) | (%Bias) | Recovery % | | (CV%) | (%Bias) |
| 0.15 | 104.26 | 0.898 | 4.26 | 96.362 | | 3.682 | -3.64 | 103.52 | | 3.255 | 3.52 |
| 1.5 | 96.214 | 4.139 | -3.79 | 104.43 | | 2.834 | 4.43 | 96.413 | | 2.266 | -3.59 |
| 2.25 | 98.325 | 1.630 | -1.67 | 97.071 | | 1.119 | -2.93 | 101.663 | | 1.453 | 1.66 |

### Table S4: Stability evaluation of ganciclovir in rabbit plasma under various storage and handling conditions using low (0.15 μg/mL) and high (2.25 μg/mL) QC samples (n=3).

| Condition | LQC (0.15 μg/mL) | | HQC (2.25 μg/mL) | |
| --- | --- | --- | --- | --- |
|  | **Recovery (%)** | **CV (%)** | **Recovery (%)** | **CV(%)** |
| Room temperature (24 h) | 96.23 | 4.52 | 97.18 | 3.87 |
| Refrigerated (2-8°C, 24 h) | 100.45 | 2.76 | 99.87 | 2.92 |
| Post-preparation (autosampler, 10°C, 48 h) | 98.65 | 3.42 | 99.34 | 3.18 |
| Three freeze-thaw cycles (-80°C) | 92.15 | 4.98 | 93.42 | 4.65 |
| Long-term storage (30 days, -80°C) | 94.87 | 4.23 | 95.34 | 3.98 |


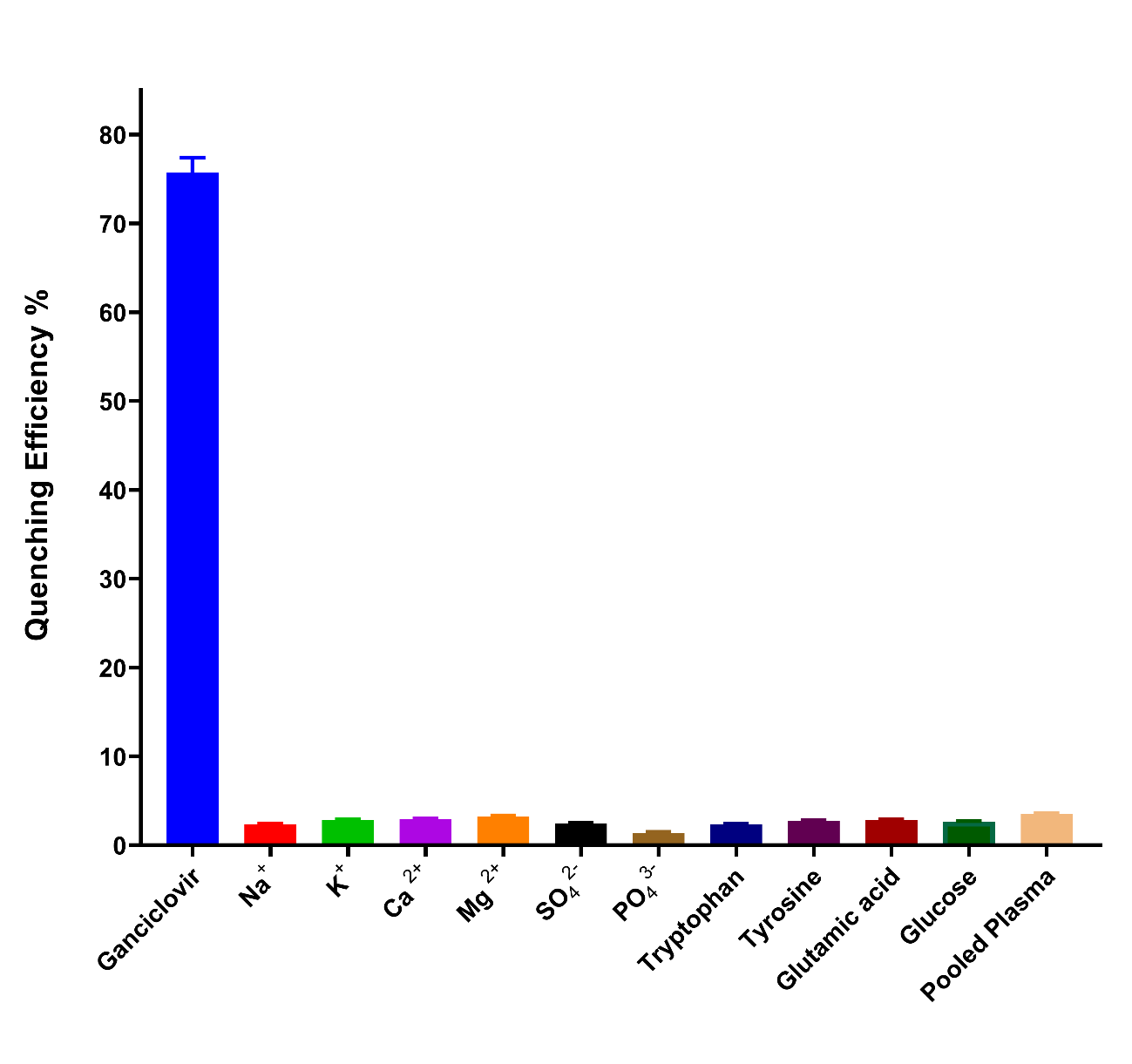


**Fig. S1:** Selectivity evaluation showing fluorescence quenching efficiency of ganciclovir compared to potential interferents including endogenous compounds, biological components, and pooled plasma.
